# Visualization of JOV abstracts

**DOI:** 10.1007/s12650-017-0451-5

**Published:** 2017-10-05

**Authors:** Koji Koyamada, Yosuke Onoue, Miki Kioka, Tomoya Uetsuji, Kazutaka Baba

**Affiliations:** 1 Academic Center for Computing and Media Studies, Kyoto University, Kyoto, Japan; 2 Science for Innovation Policy Unit, Center for the Promotion of Interdisciplinary Education and Research, Kyoto University, Kyoto, Japan; 3 Graduate School of Engineering, Kyoto University, Kyoto, Japan

**Keywords:** Review crisis, Peer review, Move analysis, Text visualization, Machine learning

## Abstract

**Abstract:**

Since the abstract can be found at the beginning of most scientific articles and is an essential part of the article, several attempts have been made to explore the rhetorical moves of abstracts in various research fields. These studies dealt only with accepted articles since they can be easily accessed. Although the findings of such works have some pedagogical implications for academic writing courses for young researchers who are relatively new to their fields, they do not contribute enough to the transparency of the peer review processes conducted in research fields. Increasing transparency requires considering rejected articles since they help to clarify the decision criteria in the peer review. Based on 591 abstracts of accepted or rejected articles submitted to *Journal of Visualization* (*JOV*), the present study aimed at exploring the differences between the accepted and rejected abstracts. The results show that there are significant differences in the structures of the abstracts. Since we also successfully develop a classification model for the decision using a machine-learning technique, the findings of this study have some implications for developing a semi-automatic reviewing system that can reduce the reviewer’s burden and increase the review quality.

**Graphical abstract:**

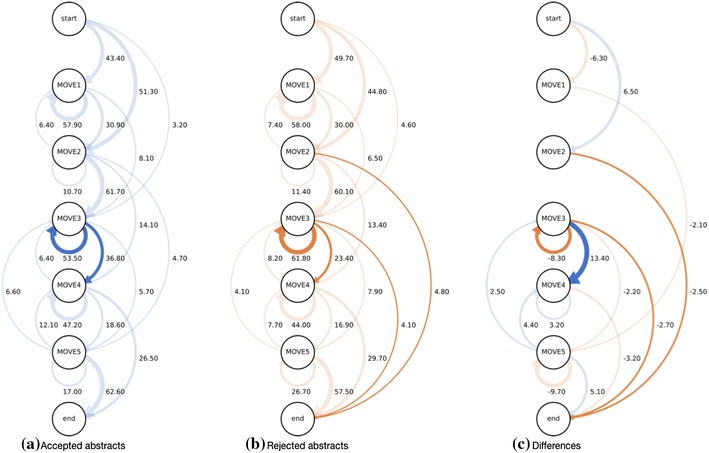

## Introduction

Since the abstract can be found at the beginning of most scientific articles and is an essential part of the article, several attempts have been made to explore the rhetorical moves (communicative functional units of a text) of abstracts in various research fields. Research article abstracts have become an important genre in any field of inquiry because they play a critical role to persuade reviewers to accept and attract readers to read the paper itself.

Jiang and Hyland examined the interactive and interactional functions of certain nouns based on rhetorical moves in 240 research abstracts from six disciplines (Jiang and Hyland 2017). They showed how these nouns are frequently employed to strategically communicate their arguments and the value of their research to the target discourse community, the use of which depends on moves and disciplines. Using 30 abstracts from three journals, Pho identified the rhetorical moves of abstracts and linguistic features that help distinguish the moves in the fields of applied linguistics and educational technology (Pho 2008). The study identified three obligatory moves in these two disciplines—presenting the research, describing the methodology, and summarizing the results, although there are two other moves that were used differently across the journals. At the same time, patterns of linguistic features in each move were similar across the journals Stotesbury reported on a study of the language used to evaluate the research based on 300 abstracts from the fields of the humanities, social and natural sciences (Stotesbury 2003). The results revealed differences in the frequency and type of explicit evaluations in the abstracts across disciplines, and across rhetorical moves for the case of modality. In a keyword extraction of patents, a technique was proposed to automatically extract such keywords that relate to novelties or inventive steps from patent claims, which are equivalent to the moves in scientific articles using the structure of the claims (Suzuki and Takatsuka 2016). The proposed technique was evaluated using rejected and granted patents.

Since the peer review process is facing a crisis, which we discuss later, it is important to increase the transparency by visualizing the abstracts of scientific articles. Peer review is the evaluation of work by one or more people of similar competence to the producers of the work (peers). It constitutes a form of self-regulation by qualified members of a profession within the relevant field. Peer review is at the heart of research and scholarly communication. Journals use it to determine what research findings are included in the scholarly record, and researchers consider the rigor of peer review important for advancing the quality of their own research and the research on which they rely to inform their work. Recent controversies highlighting substandard peer review in scientific journals have increased the need for authors, funders, publishers, and institutions to assure the quality of peer review in academic journals.

It was predicted that a significant increase in requests for reviews would be observed over time and that some reviewers would be likely to refuse a review request due to increases in reviewer burden. There is no doubt that the number of manuscripts submitted for publication in scientific journals has increased substantially in the last few decades, primarily due to an increase in the number of scientists (Grossman 2014).

The number of submitted manuscripts is related to the number of open-access journals. A study on the development of publishing of open-access journals from 1993 to 2009 (Laakso et al. 2011) suggests that, as measured by both the number of journals and the increases in total article output, direct gold open-access journal publishing has seen rapid growth, particularly between the years 2000 and 2009. It was estimated that there were approximately 19,500 articles published in open-access journals in 2000, while the number has grown to 191,850 articles in 2009. The journal count is estimated to have been 740 for the year 2000 and 4769 for 2009; these numbers indicate considerable growth, albeit at a more moderate pace than the article-level growth. These findings support the notion that open-access journals have increased both in number and in average annual output over time. The number of scientists is related to the capacities of graduate schools. Twelve Japanese universities reconstructed their education and research systems in the 1990s, following the Japanese government’s policies. We call this large change “a policy with overriding priority for the graduate school.” They expanded master’s and PhD programs, while they eliminated some departments relating to undergraduate education. The largest change is that professors’ main affiliations became the Graduate School after the reconstruction in the 1990s. It is as if the university is attached to the Graduate School. Given the increasing numbers of both journals and submissions, coupled with a pool of experienced referees that, while increasing, is still insufficient to handle the current load, it is obvious that the research community has yet to effectively deal with the ‘review crises’ (Fox and Petchey 2010).

In the visualization, the abstract is first coded using the move analysis and then converted into a set of numeric codes. A move is defined as a structural and rhetorical textual unit that has a specific communicative function and purpose (Swales 1981) and a basic element to describe a structural pattern in a scientific article. Maswana visualized all move transitions of a whole paper to show both the prototypes and the diversity of research paper textual structures using the Graphviz software (Maswana 2014). In the graph representation, nodes are connected in a sequence. They used the results of move analysis of 13 research papers in education and 8 research papers in economics. Their study was the first effort to visualize characteristics of research papers, using moves and linguistic features. The resulting graphs presented both the typical patterns and the diversity of move transitions, as well as information about language in each move. When multiple abstracts are processed in single disciplines, they expect that some features can be extracted. Previously, the visualization dealt with only accepted articles since they can be easily accessed.

Although the findings of such work have some pedagogical implications for academic writing courses for young researchers who are relatively new to their fields, they do not contribute sufficiently to the transparency of the peer review that is conducted in these fields. Increasing the transparency requires the consideration of rejected articles to clarify the decision criteria in the peer review, which will result in more pedagogical implications. How the difference between is accepted and rejected articles related to the transparency of the peer review? The present study aims at exploring the difference between the accepted and rejected abstracts based on 591 abstracts of articles submitted to *Journal of Visualization* (*JOV*).

Our research question is whether there is a difference between the structures of accepted and rejected abstracts. We expect that there is such a difference if the abstract coded by the move analysis is differently visualized according to the related decisions. In this research, the structure of the abstract is defined as both a move structure and its text contents. The move structure is coded based on move analysis described above. Also, document embedding that is a dense vector representation of texts obtained by a natural language processing (NLP) technique is used to represent the information of text contents.

The result of the comparison of move structure between accepted and rejected abstracts suggests a statistically significant difference. Then, we employ a graph-drawing technique that visualizes the abstract structure to test the hypothesis. The abstract structure in each accepted or rejected abstract exhibited different features in some parts. These results may be a clue for supporting our hypothesis. That is, the visualization of abstract structure may reflect the quality of the abstract in a submitted paper. Thus, the result suggests that the difference in abstract structure consist of the move structure and document embedding might be a strong candidate for a paper-reviewing criterion.

In addition, we derive a classification model in which the decisions can be predicted using a machine learning technique. This model classifies each paper as either accepted or rejected. Taking all the results into account, we suggest that there are differences in abstract construction between accepted and rejected papers and that visualizing the difference may be helpful in the paper-reviewing procedure to some extent. We can state that the outcome of our research work can realize transparency of the peer-review process as an indicator of peer-review quality (Wicherts 2016).

## Methods

### JOV abstracts dataset

In this research, we employed JOV articles that have been archived in the reviewing system managed by Springer. We have received approval for the use of the articles from Springer and the directors of the Visualization Society of Japan (VSJ). To ensure the protection of computer-processed personal data, all the personal data of the authors and reviewers were removed. We used the abstract text and decision data of accepted and rejected submissions only for investigating whether there is a difference between the structures of accepted and rejected abstracts.

The dataset originally contains 939 abstracts of “Regular Paper”, “Short Paper”, “Review Paper”, and “Portfolio Paper” article types. We have removed abstracts with the decision status “Withdraw” or “Under decision” from the original dataset. Then, of the remaining 847 abstracts, 591 abstracts are used in this paper.

### Visualization of move structure

To visualize their structures of the abstracts, we converted the abstract text data into a sequence of codes based on move analysis. Move analysis is one of the useful techniques for exploring the structure of text data that belong to a specific genre such as research papers and abstracts. A move is defined as a cluster of text information that has some special objective or communicative function in the context of the article. This is the method with which the texts of the papers are analyzed by categorizing the text segments according to their functions or objectives. In move analysis, usually each sentence or a group of sentences in the text is labeled to represent the structure. In many cases, move analysis was used mainly for pedagogical purposes to support novice learners to acquire genre features. In case of research papers, move analysis assisted researchers and graduate students in learning how to write research papers and provided relevant information for teachers by demonstrating how to organize arguments persuasively to the disciplinary community members (Jiang and Hyland 2017).

In this paper, all sentences in 591 abstracts, which were submitted between August 2011 and December 2016, were manually categorized and labeled into five moves. Table 1 shows the categorization of the moves and their functions. The move composition in each abstract may reflect the abstract structure.

**Table 1:** Categorization of moves in article abstracts (Jiang and Hyland 2017, p. 4)

Move	Function
1. Introduction/background	Establishes the context of the paper and motivates the research or discussion
2. Purpose	Indicates purpose, thesis or hypothesis and outlines the intention of the paper
3. Methods	Provides information on design, procedures, assumptions, approach, data, etc.
4. Results	States main findings or results, the argument, or what was accomplished
5. Conclusion	Interprets or extends results beyond the scope of the paper, draws inferences, and points to applications or wider implications

To examine the occurrence of each move, the presence or absence of the five moves in each abstract was studied and was summarized along with each decision status (accepted or rejected). Then the occurrence of each move was statically compared between the two status. The differences between the categories were assessed statistically using *t* test.

In addition, we visualize move transitions represented as a directed network with arc diagram (Wattenberg 2002). With arc diagram, we expect that the graphs will clearly show the structural features of abstracts of submitted papers. In the graphs, nodes represent moves, and the widths of path proportionally correspond to the occurrence ratio from one move to another. Prior to drawing the graphs, we first processed each abstract to construct a matrix that represents a move adjacency. Then, using the matrix, the move transition was visualized in the graph to describe the abstract structure. Finally, we compared the abstract structures generated from both the accepted and the rejected articles using the move transition graph to observe their variations.

### Visualization of document embedding

It is natural idea to apply natural language processing (NLP) approaches to illustrate the difference between accepted and rejected abstracts. To analyze the abstracts, we converted the abstract text data into numeric data using several methods. As mentioned above we use move analysis for the first conversion. Move analysis is a conversion technique since it produces a set of numeric data, the values of which range from 1 to 5. There are many other such conversion techniques that can be employed for the NLP applications.

Word2Vec is a group of related models that are used to produce word embedding (Mikolov et al. 2013). Word embedding is the collective name for a set of language-modeling and feature-learning techniques in NLP in which words or phrases from the vocabulary are mapped to vectors of real numbers. Conceptually, it involves a mathematical embedding from a space with one dimension per word to a continuous vector space of much lower dimension.

In this paper, we use Doc2Vec to convert the abstract into numeric data. Doc2Vec is a NLP method that convert a text into its dense vector representation based on neural network techniques (Le and Mikolov 2014). Doc2Vec is an extension of Word2Vec. The target of Word2Vec is a word while the target of Doc2Vec is sequence of words. The vector representation obtained by Doc2Vec is called document embedding. The document embedding maps sequence of words to relatively low dimensional space. It is known that the document embedding has good characteristics for several applications such as visualization and machine learning of documents.

Obtained document embedding of accepted and rejected abstracts are visualized two-dimensional scattered plot using t-SNE. t-SNE is a type of dimensional reduction method used for word embedding and document embedding (Maaten and Hinton 2008). To compare the vector features of accepted and rejected paper abstracts, we employ t-distributed stochastic neighbor embedding (t-SNE) technique to convert each high-dimensional vector data in a two-dimensional space. t-SNE minimizes Kullback–Leibler divergence (KL-divergence) (Kullback and Leibler 1951).

In this paper, we use Gensim (https://radimrehurek.com/gensim/) that is a NLP package for Python programming language to compute Doc2Vec. For the computation, we set the size of embedding vectors to 10. Also, we use scikit-learn (http://scikit-learn.org/) that is a machine learning package for Python programming language for t-SNE.

### Decision classification model

If there is a difference in some respects between the accepted and rejected abstracts, we can construct a classification model for decisions of the abstracts. To construct a classification model of the decision using the numeric data, we employ support vector machine (SVM) that is a well-known supervised machine learning technique. SVM classifies data that are not linearly separable based on kernel function methods (Hearst et al. 1998; Zhang 2001). In particular, we use EvoSVM that is an extension of SVM with hyper parameter optimization based on genetic algorithm (GA) (Mierswa 2006). It is important to choose optimal soft margin parameter *C* to achieve high accuracy of classification using general SVM.

We use the numerical data of the move structure and document embedding obtained by the method described in the previous sections as input of SVM. In this paper, we set the size of document embedding vector to 10; therefore, the dimension of the document embedding is 10. As mentioned in the last subsection, Doc2Vec generates relatively low dimensional vectors compared to word vocabulary size and document size. Therefore, we choose 10, which is a smaller value than the number of abstracts, as the size of vectors. The move structure includes two types of information, move occurrence and move transition. The move occurrence includes 5 values that represent the occurrence of each 5 move. Also, the move transition includes 35 values that represent possible move transition from move *X* or start to move *Y* or end. Therefore, 50 numerical values are used as feature values for constructing a classification model in total.

Generally, increasing the number of feature values of a classification model may cause over fitting problem. To avoid the over fitting, we also use a part of move structure that has statistically significant difference between accepted and rejected abstracts based on a method presented in the previous section. Finally, the combination of the input data is shown in Table 2.

**Table 2:** Description of input data

Input data	Description
A	Move structure
B	Move structure (statistically significant)
C	Document embedding
D	A + C
E	B + C

To examine the robustness of the classification model, we apply tenfold cross validation. In the tenfold cross validation, input data are divided into learning data (90%) and test data (10%). The learning data are used to estimate parameters for classification models and the test data are used to evaluate the classification accuracy of the obtained classification model. This process is repeated 10 times. Then, the average accuracy is reported as a result.

We use RapidMiner that is a freely available data science software platform (https://rapidminer.com/). In RapidMiner, users can define data processing process by using a graphical programming environment. EvoSVM and cross validation used in this paper are provided as building blocks in RapidMiner.

## Results

### Visualization of move structure

**Fig. 1:**
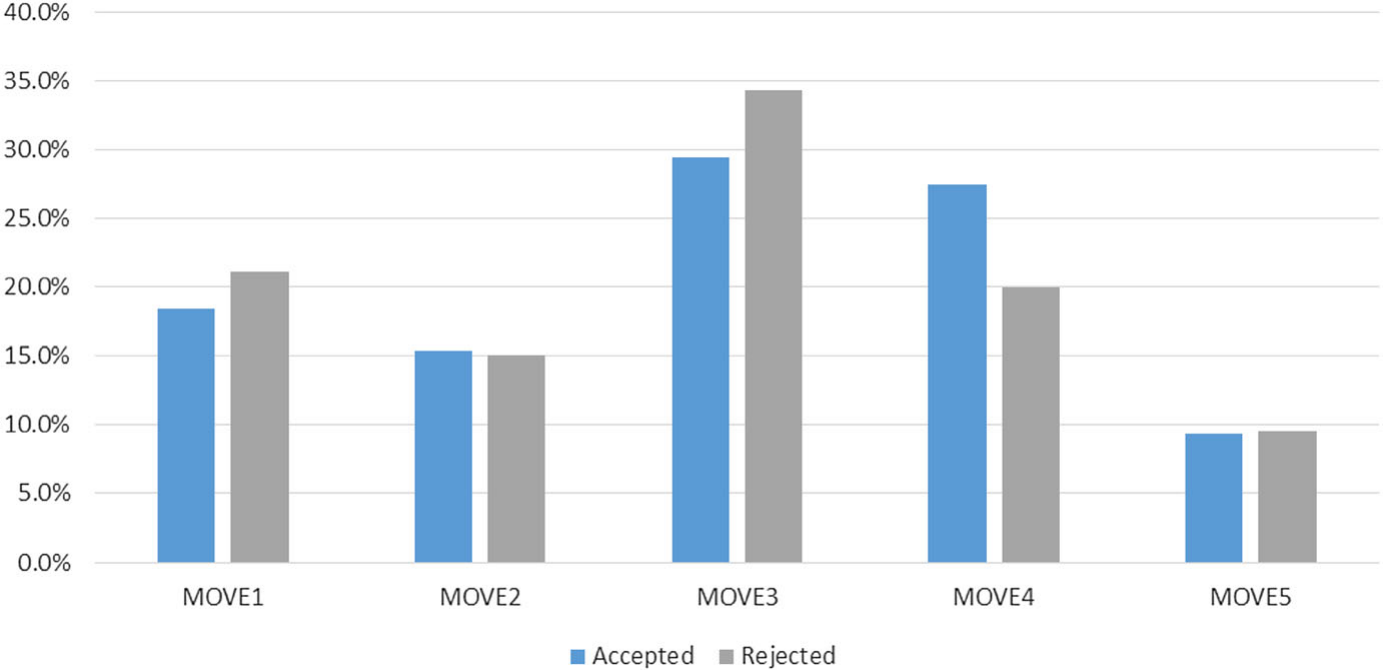
Move occurrence ratios in JOV abstracts

First, we counted the number of sentences in every move which were manually labeled from move 1 to move 5, and calculated the move occurrence ratio in abstracts of accepted/rejected papers. The move occurrence ratios in the accepted and rejected abstracts are shown in Fig. 1.

The 591 abstracts contained 4284 moves, with 19.9% containing a background move, 15.2% a purpose move, 32.1% a methods move, 23.4% a results move, and 9.5% a conclusion move. For the 272 accepted abstracts, with 1952 moves, the ratios were 18.4, 15.4, 29.5, 27.5, and 9.3%, respectively. The 319 rejected abstracts contained 2332 moves, and the ratios were 21.1, 15.1, 34.3, 20.0, and 9.6%, respectively.

In the accepted group, move 4 makes up 27.5% of all moves, which is obviously higher than that in the rejected group. Conversely, the rejected group has more move 1 and move 3 sentences than the accepted group. These results indicate that the accepted abstracts contain longer descriptions of the results than the rejected abstracts, which contain longer descriptions of background and methods.

Table 3 shows the result of paired *t* test of the move occurrence. The difference in frequency of move 1, 3, and 4 between the accepted and rejected abstracts was confirmed to be statistically significant. This shows that there is a significant difference in move occurrence pattern between the two groups.

**Table 3:** Result of *t* test of move occurrence

Move	*p*	*t* value
1	0.016*	2.427
2	0.505	0.667
3	0.025*	2.247
4	>0.001*	4.310
5	0.478	0.711

* Means the existence of statistically significant difference

**Fig. 2:**
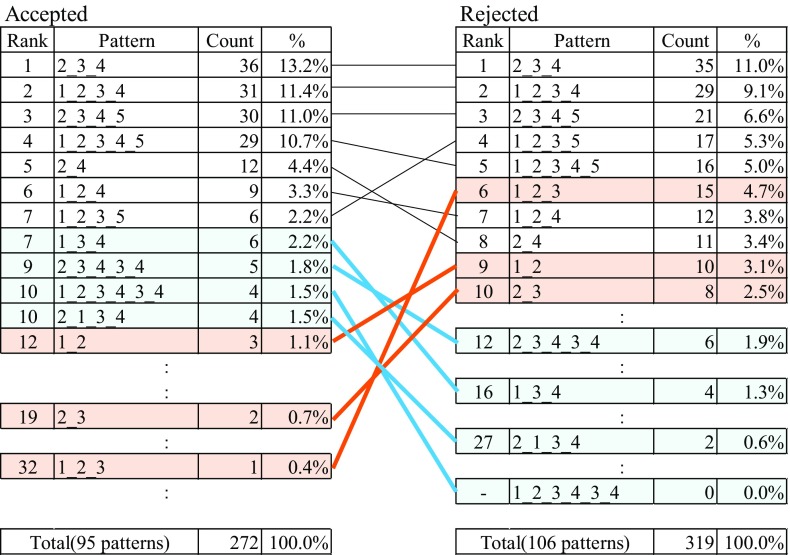
Move transition of accepted and rejected JOV papers

Figure 2 describes the move transitions in the accepted and rejected abstracts and their occurrence ratio (%). We analyzed the transition of the moves that appeared in each abstract and ranked the patterns in descending order. We chose the top 10 ranks of move transitions from accepted and rejected abstracts, respectively. The same transition patterns are connected with a bar and patterns found only in either one’s top 10 rank are marked with color (blue for accepted, orange for rejected groups, respectively).

Among the top 5 move transitions, four patterns are shared by the accepted and rejected groups. They are 2 → 3 → 4, 1 → 2 → 3 → 4, 2 → 3 → 4 → 5, and 1 → 2 → 3 → 4 → 5, the occurrence ratio are 13.2, 11.4, 11.0, and 10.7%, respectively in accepted group. And in the rejected group, each ratio are 11.0, 9.1, 6.6, and 5.0%, respectively. This suggests that most of the move transitions found in JOV paper abstracts show relatively straight forward pattern in both groups.

On the other hand, three or four different move transition patterns are observed. In the accepted group, four patterns of 1 → 3 → 4, 2 → 3 → 4 → 3 → 4, 1 → 2 → 3 → 4 → 3 → 4, and 2 → 1 → 3 → 4 are among the top 10 of the accepted abstracts (marked blue), and amounted to 7% but in the rejected group, they are found out of the top 10 patterns and occurred collectively only 3.8%. Meanwhile, for the rejected group, we find 1 → 2 → 3, 1 → 2 and 2 → 3 among the top patterns (orange). The occurrence ratio of these three patterns amounts to 10.3%, in contrast to 2.2% in the accepted group. These results suggest that there is some difference in move transition patterns and that some of the rejected abstracts lack clear explanations of the results or conclusion of the paper. The comparison of the overall structures of abstracts from the JOV submitted papers indicates that there are some differences in terms of collective structure between the accepted and rejected categories.

**Fig. 3:**
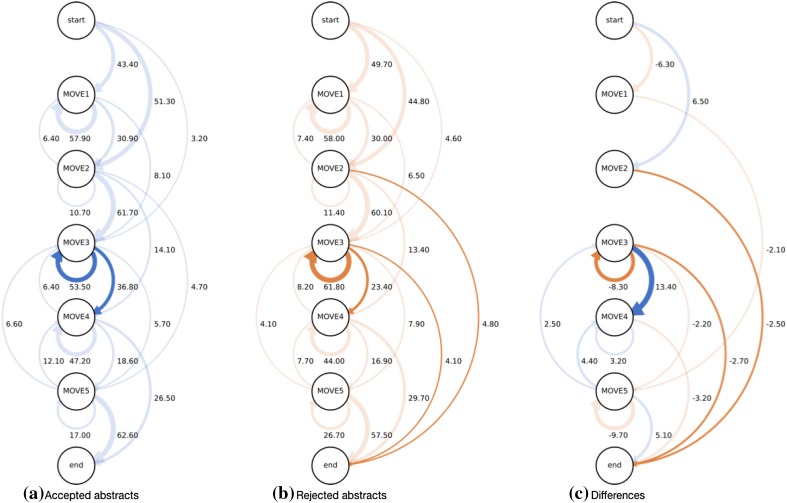
Arc diagrams of move transition. **a** Move transition of accepted abstracts. **b** Move transition of rejected abstracts. **c** The difference of the transition ratio between accepted and rejected abstracts

Figure 3 illustrates the move transition in abstracts described by arc diagram. (a) and (b) describe the move transition of abstracts in accepted and rejected JOV submitted papers. (c) summarizes the difference of the transition occurrence ratio between them. The graphs consist of nodes and paths. Each node represents start, moves, and the end and each path means the transition from one move to another. All graphs have seven nodes: the start, move 1 to move 5, and the end. Each of the numbers in the graphs indicates the transition ratio (%) from one node to another. In addition, the widths of a path proportionally correspond to the occurrence ratio in (a) and (b) or the difference of the ratio between the accepted and rejected groups in (c) in each move transition. We only draw paths with 3% or more occurrence rate in (a) and (b). In (c), only the paths with two or larger ratio (%) of the absolute difference. The paths with significant difference are shown in dark color (paired *t* test, *p* < 0.05). In (c), the dark blue paths show larger occurrence ratio in the accepted group than that in the rejected group and the orange paths vice versa.

Comparing the move transitions of the accepted and rejected groups, some difference is observed. First, in the accepted group, move transition from 3 to 4 was 13.4% higher than in the rejected group. In addition, the transition from move 3 to move 3 itself was 61.8% in rejected group and 53.5% in accepted group, and the difference of 8.3% was significantly higher in rejected group (graph c). Moreover, the transition from move 2 to end and from move 3 to the end of the rejected group are higher than those of accepted group by 2.5 and 2.7% respectively. The last two transitions suggest that some of the rejected abstracts ended at move 2 or 3.

### Visualization of document embedding

**Fig. 4:**
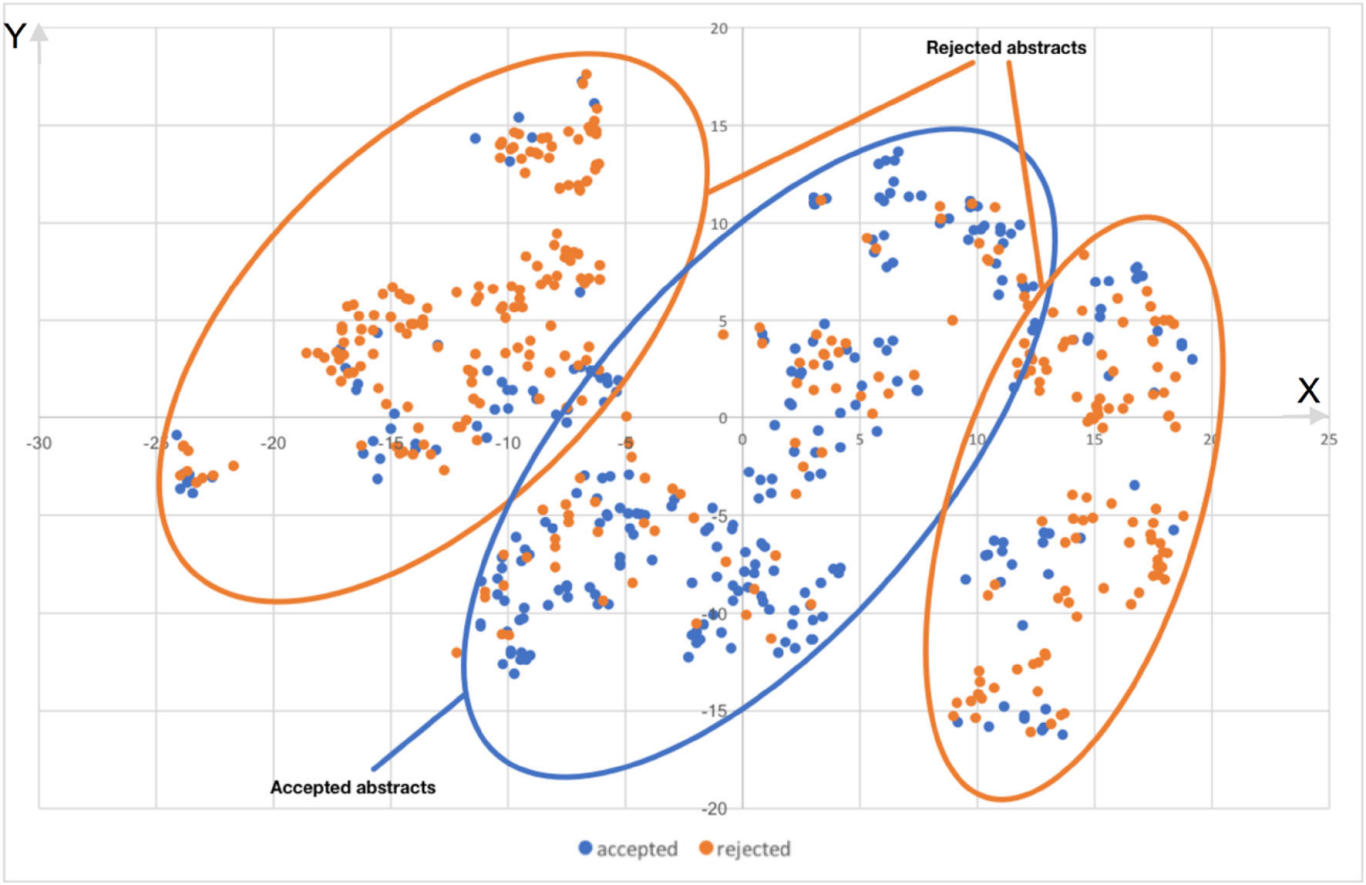
Two-dimensional scattered plot of document embedding of accepted (blue) and rejected (orange) abstracts

Figure 4 shows two-dimensional scattered plot of document embedding obtained by Doc2Vec and t-SNE. In Fig. 4, optimized KL-divergence is 0.75. Each point in Fig. 4 represent accepted (blue) and rejected (orange) abstracts.

From Fig. 4, it seems that the abstracts were classified into some clusters. There are two types of clusters, a cluster containing many accepted abstracts and a cluster containing many rejected abstracts. In Fig. 4, the area surrounded by blue and orange line contains many accepted and rejected abstracts, respectively.

We examined the text contents in each cluster and found that abstracts in the center cluster have move 4 with 20% higher rate than those in right or left cluster. In fact, 89.8% of the abstracts in the center cluster have move 4, while, 68.1 and 70.8% of them in the right and left clusters, respectively. This means most of abstracts in the center cluster clearly described results of the research.

The original vector dimension is 10; therefore, the drawing result does not completely reflect the relative position in the high dimensional space. However, the abstracts contained in the same cluster tend to be placed near in the high dimensional space.

### Decision classification model

We applied SVM to classify the accepted the rejected abstracts. Table 4 shows the results of tenfold cross-validation with 5 input data shown in Table 2. In C, D, and E, the classification accuracy depends on vector value generated by Doc2Vec. Therefore, average, maximum, and minimum accuracy of 20 times trials are presented in Table 4.

**Table 4:** Accuracy of the decision classification model using SVM

Input	Accuracy		
	Avg.	Max.	Min.
A	53.54	53.54	53.54
B	56.70	56.70	56.70
C	76.06	78.67	73.69
D	76.81	78.68	73.72
E	77.71	80.01	76.03

The classification accuracy of A and B, which are move transition and its statistically significant subset, are 53.54 and 56.70. The accuracy of B is slightly higher than the accuracy of A. However, both of them are relatively low. The result shows that it is difficult to classify the decision using only move transition information by SVM.

In C, the average classification accuracy using the document embedding vectors is 76.06. The accuracy of C is clearly higher than the accuracy of A and B. In addition, input data combining move transition and document embedding, D and E, further improve the classification accuracy. In the best case, the classification accuracy of E exceeds 80%. It is confirmed that accurate classification model can be construct with both move transition and document embedding of the abstracts.

It is also confirmed that the classification accuracy of B and E is higher than A and D, respectively. This means that using statistically significant subset of move transition achieves higher classification accuracy than using all of move transition with SVM.

## Discussion

As described in the previous section, it is confirmed that there are differences in the trends of the move transitions between the accepted and rejected abstracts. In addition, the accepted abstracts contain more result and discussion moves than the rejected papers. On the other hand, the rejected abstracts contain more method moves and fewer result and discussion moves (Fig. 1). This result implies that research contributions described as result and discussion moves should be emphasized in the abstracts. This result is not in contradiction with knowledge in teaching research-paper writing. The result is also supported by Fig. 2. In Fig. 2, all the top 10 patterns of the accepted abstracts include at least move 4 or 5, however, those of the rejected abstracts sometimes lack move 4 and/or 5. This indicates that some of the rejected abstracts lack clear explanations of the results or conclusion of the paper.

The move transitions of both accepted and rejected abstracts are visualized as directed graphs (Fig. 3). It is confirmed that there are differences between the graphs of accepted and rejected abstracts in some parts. The visualization of move transitions will be helpful in understanding the differences between the structures of accepted and rejected papers. The visualization of the abstract move transition suggests that the simplicity of the foreword, sufficient volume of the result section, and straightforward conclusions might have some impact on the decision of reviewing the papers for acceptance/rejection.

The move transitions can be used as decision criteria for the peer review. Currently, most reviewers do not have clear criteria for their decisions. Thus, they decide whether a paper is accepted or not based on their implicit criteria. As a result, the decisions may depend on the reviewers, and review quality will vary. It is valuable that some of the implicit review criteria can be described as differences of move transitions. This result can be reflected in guidelines for clear review criteria.

The document embedding vectors reflect the difference of the subject but not the difference of the acceptance status. Since the word vectors provided by Doc2Vec were generated from the 591 abstracts of JOV submitted papers, this makes the corpus relatively small size and may have some influence on the feature of the word vectors. In the future, we would like to investigate the effect of the corpus size by using vectors generated from much larger text corpus such as variety of academic papers and others. In addition, we need to find optimal parameters for Doc2Vec such as the size of document embedding vector to improve the accuracy.

We have also demonstrated that machine learning approaches have the potential to predict whether a paper will be accepted. The reason why SVM for Doc2Vec result seem to work well may that the feature of space containing the document embedding vectors. The document embedding vectors generated from Doc2Vec reflect much more information such as all words, phrases, and sentences and their syntactic relations. Still, the accuracy of the proposed classification model is not very high because we have used only part of the information about the papers. Additionally, we have used only general machine learning software in this paper. It is expected that the accuracy will be improved by using more effective machine learning models that have been tuned for this purpose.

Developing machine learning models will reduce the burden of peer review and increase the review quality. According to previous studies (Maswana 2014; Jang and Hyland 2017), there are differences in the trends of move transitions among different academic disciplines. One limitation of this study is that we have used papers submitted to only one academic journal. The generality of the results should be examined in future work. However, we believe that machine learning approaches will improve the quality of the research papers published in various academic journals.

One possible future direction to improve the accuracy is to use not only the abstracts but also the body texts of the papers. It is expected that there are stronger differences in move transitions between accepted and rejected papers in the body text than in the abstract. However, it is difficult to classify all sentences contained in the body text into moves. To resolve this problem, we will develop another machine learning model to classify sentences into moves. Using the body text to realize semi-automatic reviewing is a natural idea because authors can easily take measures to exploit semi-automatic reviewing if only abstracts are used in the classification. Authors may also be able to take measures to exploit semi-automatic reviewing using the body text; however, this will improve the quality of paper.

Finally, we summarize our work. In this paper, we have investigated the differences between the accepted and rejected papers submitted to the *Journal of Visualization*. We have applied move analysis to 591 abstracts. As a result, it is clarified that there are significant differences between the move transitions of accepted abstracts and those of rejected abstracts. Additionally, it is demonstrated that the move transitions can be used as feature values of classification models for paper acceptance. In future work, we will develop accurate classification models by using various feature values, including word embedding and move transitions of the body text, to resolve the peer-review crisis.
